# Antibiotic Resistance of *Salmonella* spp. Isolated from Shrimp Farming Freshwater Environment in Northeast Region of Brazil

**DOI:** 10.1155/2013/685193

**Published:** 2013-12-22

**Authors:** Fátima C. T. Carvalho, Oscarina V. Sousa, Edirsana M. R. Carvalho, Ernesto Hofer, Regine H. S. F. Vieira

**Affiliations:** ^1^Laboratório de Microbiologia Ambiental e do Pescado, Instituto de Ciências do Mar (LABOMAR), Universidade Federal do Ceará, Avenida Abolicão 3207, CEP 60165-081, Fortaleza, CE, Brazil; ^2^Laboratório de Zoonoses Bacterianas, Instituto Oswaldo Cruz (FIOCRUZ)-Pavilhão Rocha Lima, Avenida Brasil 4365, CEP 21040-360, Manguinhos-Rio de Janeiro, RJ, Brazil

## Abstract

This study investigated the presence and antibiotic resistance of *Salmonella* spp. in a shrimp farming environment in Northeast Region of Brazil. Samples of water and sediments from two farms rearing freshwater-acclimated *Litopenaeus vannamei* were examined for the presence of *Salmonella*. Afterwards, *Salmonella* isolates were serotyped, the antimicrobial resistance was determined by a disk diffusion method, and the plasmid curing was performed for resistant isolates. A total of 30 (16.12%) of the 186 isolates were confirmed to be *Salmonella* spp., belonging to five serovars: *S*. serovar Saintpaul, *S*. serovar Infantis, *S*. serovar Panama, *S*. serovar Madelia, and *S*. serovar Braenderup, along with 2 subspecies: *S. enterica* serovar houtenae and *S. enterica* serovar enterica. About twenty-three percent of the isolates were resistant to at least one antibiotic, and twenty percent were resistant to at least two antibiotics. Three strains isolated from water samples (pond and inlet canal) exhibited multiresistance to ampicillin, tetracycline, oxytetracycline, and nitrofurantoin. One of them had a plasmid with genes conferring resistance to nitrofurantoin and ampicillin. The incidence of bacteria pathogenic to humans in a shrimp farming environment, as well as their drug-resistance pattern revealed in this study, emphasizes the need for a more rigorous attention to this area.

## 1. Introduction

The growing demand for shrimp on the international market has boosted inland shrimp farming over the past decades. Marine shrimp culture located miles away from the ocean is a new and fast growing sector of aquaculture [1]. However, shrimp farmed in both salt water and freshwater are becoming increasingly vulnerable to bacterial infection due to the ease with which pathogens are transmitted in aquaculture [2]. As a result, many farmers have made improper use of antibiotics to prevent or treat infections, leading to the dissemination of antimicrobial-resistant strains in aquatic environments [3]. The increase in the incidence of antimicrobial-resistant strains is also associated with the presence of plasmids containing resistance genes, providing microbiological populations with a greater genetic flexibility and allowing them to adapt and survive in hostile environments [4]. The addition of antibiotics to shrimp fodder for prophylaxis, treatment of infections, or growth stimulation has contributed to the perpetuation of resistant and pathogenic strains as well [5].

Shrimp farm workers are not only regularly exposed to antibiotics as these are mixed with the fodder, but they are also vulnerable to contamination by multiresistant invasive bacteria, such as *Salmonella *spp. Patients with *Salmonella* infection should be treated with antibiotics that are effective against these bacteria [6, 7].

The objectives of this study were to investigate the presence of *Salmonella* strains in shrimp farm environmental samples and to determine the antimicrobial resistance profiles among isolated strains from ponds and inlet canals of shrimp farms in Northeast Region of Brazil.

## 2. Materials and Methods

### 2.1. Sampling

Samples were collected at two farms (A and B) rearing freshwater-acclimated *Litopenaeus vannamei* located in the estuary of the Jaguaribe River (Jaguaruana City, State of Cear, Brazil). A total of hundred and twenty samples were collected from shrimp farms in two time periods: from June to December 2007 and from June to September 2008. The number of samples was the same in both periods, consisting of water (30) and sediment (30), from ponds and inlet canals.

Sampling covered the complete crop cycle. Water (2 L) was collected in sterile amber vials and filtered *in loco* through a many-fold layer (five) of sterile gauze. Gauze was then immersed in 225 mL lactose broth and incubated for 24 hours at 35°C.

Each sediment sample consisted of four subsamples from random locations inside the pond or inlet canal pooled into a single homogeneous sample. A 25 g aliquot was inoculated in 225 mL lactose broth and incubated for 24 hours at 35°C.

### 2.2. Microbiological Analysis

Samples were processed for the presence of *Salmonella* by using the 3-step technique described by Wallace and Hammack [8]: enrichment with Rappaport-Vassiliadis broth and Tetrathionate broth; plating onto selective media; Hektoen enteric agar; and MacConkey agar (all Difco). Suspicious *Salmonella* colonies were selected from the plating media and transferred to trypticase soy agar (TSA), triple sugar iron (TSI) agar, lysine iron agar (LIA), and sulfide indole motility (SIM) agar (all Difco). Isolates with typical reactions were biotyped for identification of *Salmonella *species and subspecies according to Le Minor and Popoff [9], and they were serotyped (serogroups and serovars) using O and H antisera agglutination tests, Oswaldo Cruz Institute, RJ, Brazil [9].

### 2.3. Antimicrobial Susceptibility Testing

The antimicrobial susceptibility was performed by disk diffusion method [10] using commercially available antibiotic-containing disks (LABORCLIN, Brazil): ampicillin (AMP; 10 *μ*g), tetracycline (TET; 30 *μ*g), gentamicin (GEN; 10 *μ*g), chloramphenicol (CHO; 30 *μ*g), florfenicol (FLF; 30 *μ*g), ciprofloxacin (CIP; 5 *μ*g), nitrofurantoin (NIT; 300 *μ*g), and nalidixic acid (NAL; 30 *μ*g). The oxytetracycline disks (OTC; 30 *μ*g) were prepared using blank disks (6 mm, LABORCLIN) impregnated with the antibiotic solution [11]. These antibiotics were tested according to their use in medicine, veterinary medicine, and farming for the prevention and treatment of diseases in Brazil. For control of test, the following reference strains were used: *Salmonella* ser. Choleraesuis ATCC10708, *Escherichia coli* ATCC25922, *Pseudomonas aeruginosa* ATCC27853, and *Staphylococcus aureus* ATCC25293.

The breakpoints considered for florfenicol and oxytetracycline, two antibiotics commonly used in shrimp farming to prevent and treat infectious disease outbreaks, were the same for chloramphenicol and tetracycline disks, respectively, according to CLSI [11].

### 2.4. Plasmid Curing

Strains resistant to more than one antibiotic were submitted to plasmid curing as described by Molina-Aja et al. [12], using Luria-Bertani broth (Difco) supplemented with 0.85% NaCl and 100 *μ*g/mL of acridine orange dye. Susceptibility was tested against antibiotics after the process.

### 2.5. Determination of the Inhibitory and Bactericidal Concentrations

Minimum inhibitory concentration (MIC) and minimum bactericidal concentration (MBC) were determined in strains resistant to antimicrobial test disk diffusion (Kirby-Bauer) according to the broth dilution (macrodilution) technique using Mueller-Hinton broth, Difco CLSI [11].

## 3. Results

*Salmonella* spp. accounted for 30 (16.12%) of the 186 suspected strains. Five serovars were identified: *S*. ser. Saintpaul (*n* = 2), *S*. ser. Infantis (*n* = 6), *S*. ser. Panama (*n* = 3), *S*. ser. Madelia (*n* = 6), and* S*. ser. Braenderup (*n* = 5), along with two *S. enterica *subspecies: houtenae (*n* = 6) and enterica (*n* = 2). Most *Salmonella *strains came from Farm B (*n* = 24/80%) (Table 1).

Six of the *Salmonella* strains were resistant to one or more of the following antibiotics: AMP, OTC, TET, and NIT. Among the isolates, three multidrug-resistent strains (Sw15, Ss16, and Ss17) displayed resistance to three antibiotic classes (penicillin, tetracycline, and nitrofuran). A strain of *Salmonella *ser. Panama (Sw11) was susceptible to all antibiotics in the antibiogram test. Isolates colonies (subpopulation, strain Sw11a) grew within the inhibition zone of tetracycline disks (TET and OTC) (Table 1).

The results of the plasmid curing revealed that most of the antibiotic-resistant *Salmonella* expressed resistant phenotype after their incubation with mutagenic agent (acridine orange), indicating that this profile is probably encoded by chromosomal genes. Only one cured strain of *Salmonella* (Ss17) lost its resistance towards AMP and NIT, indicating that these genes are related to plasmids.

MIC for tetracyclines (TET and OTC) ranged between 32 *μ*g and 64 *μ*g for strains isolated from river water on Farm A (Sw3) and from inlet canal water (Sw10, Sw11, and Sw15) and sediment on Farm B (Ss16 and Ss17). For tetracyclines, MBC ranged between 64 *μ*g and 1024 *μ*g (Sw10 and Sw11a*). MIC and MBC for AMP and NIT were above 640 *μ*g/mL (Table 2).

## 4. Discussion

The presence of *Salmonella* spp. in coastal and inland shrimp culture environments in Northeast Region of Brazil has been recorded by several authors [13–15]. The presence of these bacteria is primarily related to the discharge of untreated sewage into local water bodies and also to the reuse of water resources, such as organic fertilizers for irrigation and aquaculture [16].

In a study on shrimp culture environments and livestock in India, Bhaskar et al. [1] found *Salmonella* in all samples, but concentrations were poorly correlated with bacterial indicators of fecal contamination. This could suggest that *Salmonella* belong to autochthonous microbiota. According to Reilly et al. [17], pond fertilization with untreated manure is a major source of *Salmonella *contamination in shrimp farm sediments. This practice, however, is not adopted by Brazilian shrimp farmers; in fact, according to Brito et al. [18], ponds in Northeast Region of Brazil are fertilized mainly with urea and triple superphosphate, and less frequently with nitrates, phosphates, and sulfates. The presence of *Salmonella* in Brazilian aquaculture is therefore attributed to the presence of sewage discharge and/or grazing animals in the area surrounding the farm.

Salmonellosis, a major concern in worldwide public health, is characterized by high levels of endemicity and morbidity, and it is particularly difficult to control due to a variety of epidemiological parameters related to multiple sources of infection and routes of transmission throughout the cycle of the disease [19]. In Brazil, fifteen food-related salmonellosis outbreaks were officially registered between 2000 and 2008 related with a wide variety of food sources [20].

The results of this study have disclosed two public health problems: (1) the presence of resistant *Salmonella *strains in Northeast Brazilian shrimp culture and (2) the possibility of *Salmonella *infection in humans through the consumption of contaminated shrimp, along with economic losses as a result of reduced exports to international markets with high standards of control of pathogens in productive and processing systems.

The isolation of antibiotic-resistant strains might suggest the use of drugs in that aquatic environment. Continuous and improper use of this antibiotic may increase the incidence and resilience of resistant strains, thereby compromising human food safety and creating a difficulty in treating human salmonellosis. In many countries, the irresponsible use of antibiotics in animal husbandry is considered a major cause of increased bacterial resistance [21–23]. In fact, Cabello [24] discussed the effect of selective pressure of antibiotic drug residue on the microbial composition in aquaculture pond sediments.

The binding of antibiotic to sediment particles delays its biodegradation and explains long-term permanence of the drugs in the environment. Drugs of the NIT group have been banned since 2003 from veterinary practice and food production (Ministry of Agriculture, Norm no. 09) [25], and NIT-resistant bacteria are still isolated in shrimp farm sediments. It was possible to verify resistance to high concentrations of antibiotics (NIT and AMP) among the *Salmonella* spp. isolated in Farm B from both water and sediment samples (Table 2).

It should be noted that OTC-resistant *Salmonella* strains were also observed in this study. In fact, this antibiotic is widely used by shrimp farmers in the region to the treatment of bacterial disease in shrimp culture [26]. Due to low absorption, the recommended dose is 5–10 times greater than the average dose used in medical care. Thus, most OTC administered in aquaculture inevitably accumulates in pond sediments [27]. Tetracyclines and oxytetracyclines have binding sites for metal ions, especially magnesium and calcium [28]. When tetracyclines are administered in salt water, ion-binding can reduce biological activity [29]. The shrimp farms sampled for this study were located in a freshwater environment, but the inlet water (from the Jaguaribe River) was moderately hard, indicating a relative abundance of calcium and magnesium ions [30]. In other words, the hardness of the freshwater used on the shrimp farms in the study could be interfering with the action of tetracyclines.

To conclude, the results in the present study revealed the presence of *Salmonella *strains resistant to antimicrobials in cultured freshwater-acclimated *L. vannamei* farms. The incidence of bacteria pathogenic to humans in a shrimp farming environment, as well as their drug-resistance pattern revealed in this study, emphasizes the need for a more rigorous attention to this area.

## Figures and Tables

**Table 1:** Antimicrobial resistance profile of subspecies and serovars of *Salmonella enterica* isolated from water and sediment samples from ponds and inlet canal (Jaguaribe River) of two freshwater-acclimated *Litopenaeus vannamei* culture farms (A and B), Ceara, Brazil.

Origin	Source	Sample	Isolates	Subspecies-Serovars	Resistance profile
Farm A	Pond	Water	Sw1	*S*. ser. Saintpaul	—
		Sediment	—	—	—
	Inlet canal	Water	Sw2	*S*. ser. Saintpaul	—
			Sw3	*S*. ser. Madelia	OTC, TET
			Sw4	*S. enterica* subsp. houtenae	—
			Sw5	*S. enterica* subsp. houtenae	—
			Sw6	*S. enterica* subsp. houtenae	—
		Sediment	—	—	—

Farm B	Pond	Water	Sw7	*S*. ser. Infantis	—
			Sw8	*S*. ser. Panama	—
			Sw9	*S*. ser. Infantis	—
		Sediment	—	—	
	Inlet canal	Water	Sw10	*S*. ser. Panama	AMP
			Sw11	*S*. ser. Panama	—
			Sw11a*	*S*. ser. Panama	TET, OTC
			Sw12	*S*. ser. Madelia	—
			Sw13	*S*. ser. Madelia	—
			Sw14	*S*. ser. Madelia	—
			Sw15	*S*. ser. Madelia	AMP, NIT, OTC, TET
		Sediment	Ss16	*S*. ser. Braenderup	AMP, NIT, OTC, TET
			Ss17	*S*. ser. Braenderup	AMP, NIT, OTC, TET
			Ss18	*S*. ser. Braenderup	—
			Ss19	*S*. ser. Braenderup	—
			Ss20	*S*. ser. Braenderup	—
			Ss21	*S. enterica* subsp. enterica	—
			Ss22	*S. enterica* subsp. enterica	—
			Ss23	*S*. ser. Infantis	—
			Ss24	*S*. ser. Infantis	—
			Ss25	*S*. ser. Infantis	—
			Ss26	*S. enterica* subsp. houtenae	—
			Ss27	*S. enterica* subsp. houtenae	—
			Ss28	*S*. ser. Infantis	—
			Ss29	*S*. ser. Madelia	—
			Ss30	*S*. ser. Madelia	—

Sw: water-sample *Salmonella*; Ss: sediment-sample *Salmonella*; Sw11a*: subpopulation.

**Table 2:** Minimum inhibitory (MIC) and bactericidal (MBC) concentrations of antibiotics tested against resistant *Salmonella* spp. strains isolated from water and sediment samples of ponds and inlet canal (Jaguaribe River) in two freshwater-acclimated *Litopenaeus vannamei* culture farms (A and B), Ceara, Brazil.

Location	Sample	Isolates	Concentration (*μ*g)							
			Tetracycline				Penicillin		Nitrofuran	
			TET		OTC		AMP		NIT	
			MIC	MBC	MIC	MBC	MIC	MBC	MIC	MBC
Farm A	Inlet canal	Sw3	64	256	64	128	—	—	—	—

Farm B	Inlet canal	Sw10	64	64	64	64	640	>640	—	—
		Sw11a*	64	256	128	1024	—	—	—	—
		Sw15	128	256	64	512	>640	>640	6400	>12800
		Ss16	64	256	64	512	>640	>640	6400	>12800
		Ss17	64	256	64	512	>640	>640	12800	>12800

Sw: water-sample *Salmonella*; Ss: sediment-sample *Salmonella; *—: test not performed for this antibiotic.

*Subpopulation.
